# An Unusual Presentation of Hydatid Disease in a Child: A Case Report

**DOI:** 10.1055/s-0041-1731445

**Published:** 2021-06-23

**Authors:** Kamal El Haissoufi, Abdelouhab Ammor, Imane Kamaoui, Houssain Benhaddou

**Affiliations:** 1 Department of Pediatric Surgery “A,” Mohammed VI University Hospital, Oujda, Morocco; 2 Faculty of Medicine and Pharmacy, Mohammed Ist University, Oujda, Morocco; 3 Department of Radiology, Mohammed VI University Hospital, Oujda, Morocco

**Keywords:** hydatid cyst, subcutaneous, abdominal wall, lung, child, case report

## Abstract

The subcutaneous localization of the hydatid cyst in the abdominal wall is rarely encountered particularly in the pediatric population and is sometimes difficult to diagnose preoperatively. Here, we report the case of a 6-year-old boy who presented with two isolated episodes of low abundant hemoptysis and in whom a mass on the right lumbar region already considered as a lipoma was studied. Laboratory and radiological examinations were requested and the parietal cyst was surgically managed. The macroscopic and the pathological examination confirmed the diagnosis of the hydatid disease and helped in identifying the nature of the thoracic lesion that disappeared spontaneously after two episodes of hydatid vomiting. Hydatid cyst should be considered as a diagnosis for any masses of the abdominal wall. Moreover, biopsy and partial resection of the mass must be avoided.

Echinococcosis or hydatid disease is defined as a parasitic zoonosis in which the *Echinococcus cestode* worms are the causative agents. 1 There are two forms of the disease for which cystic echinococcosis (CE) and alveolar echinococcosis (AE) are pathogenic for humans. 1 CE is caused by *Echinococcus granulosus* and is the most common presentation. 1 The two most involved organs are the liver and the lung. 2 The uncommon localizations such as soft tissues including abdominal walls are rarely seen even in endemic countries especially in children. 2 Only five cases with similar localization in children were reported. 3 4 5 6 7

We report a rare case of a child with subcutaneous hydatid cyst located in the lumbar region of the abdominal wall that its diagnosis was challenging in our clinical setting. The patient was successfully managed in our tertiary academic care center.

## Case Report

**Fig. 1:**
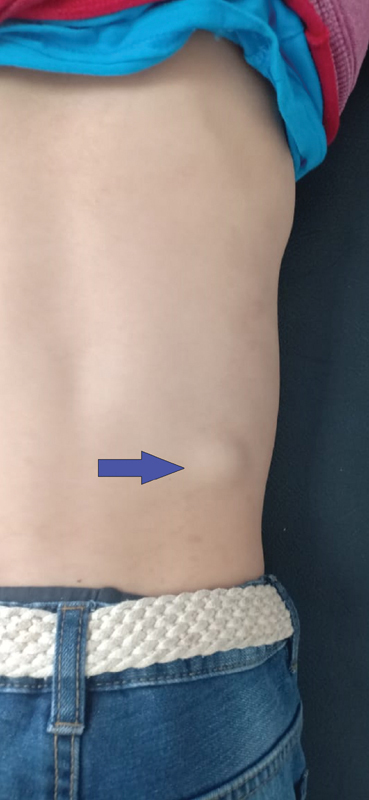
Image showing swelling of the right lumbar region (arrow).

**Fig. 2:**
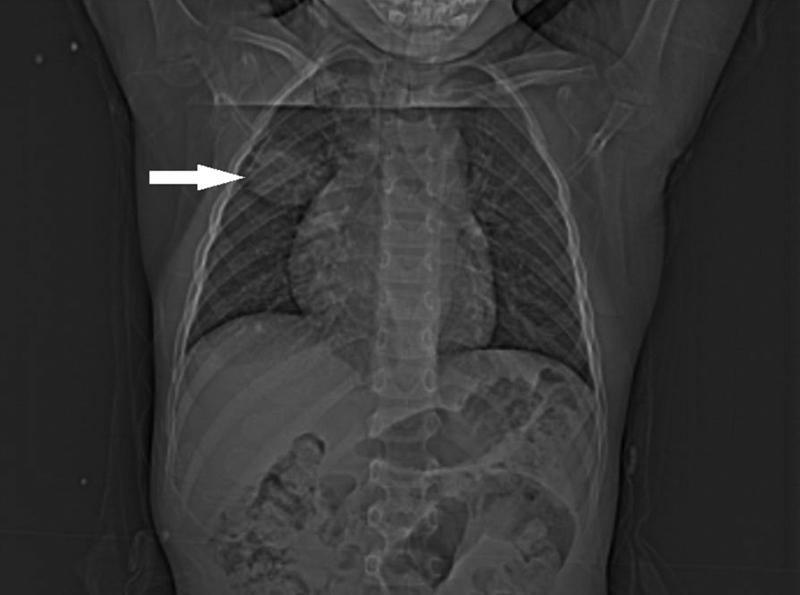
Chest X-ray image demonstrating a right middle pulmonary opacity (arrow).

**Fig. 3:**
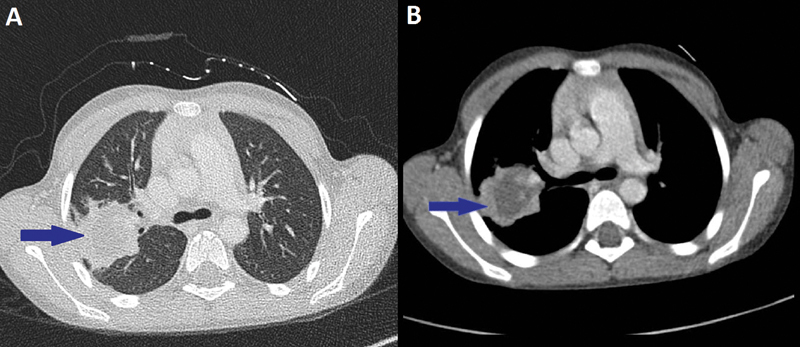
Chest computed tomographic scan showing a right cystic pulmonary parenchymal mass (arrow) in both lung window ( **A** ) and mediastinal window ( **B** ).

**Fig. 4:**
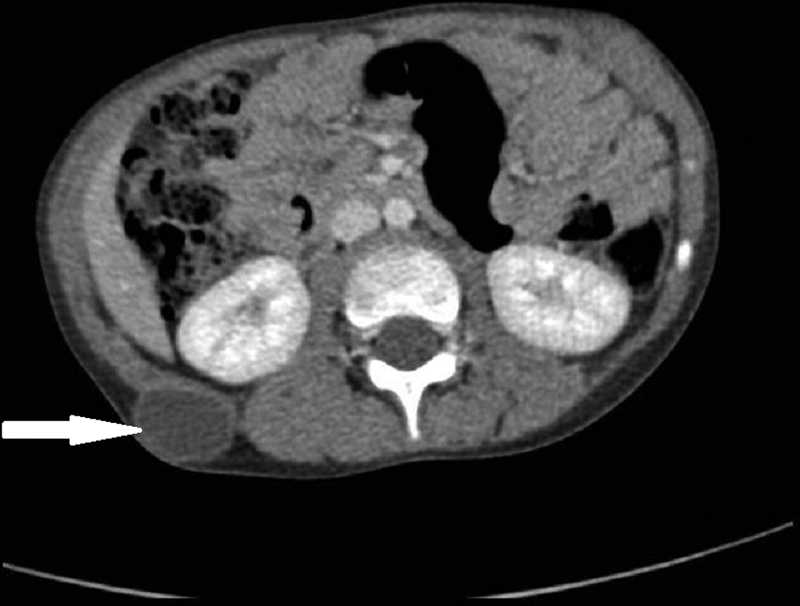
Abdominal computed tomographic scan showing a well-defined cystic mass of the subcutaneous soft tissues of the right posterolateral abdominal wall (arrow).

**Fig. 5:**
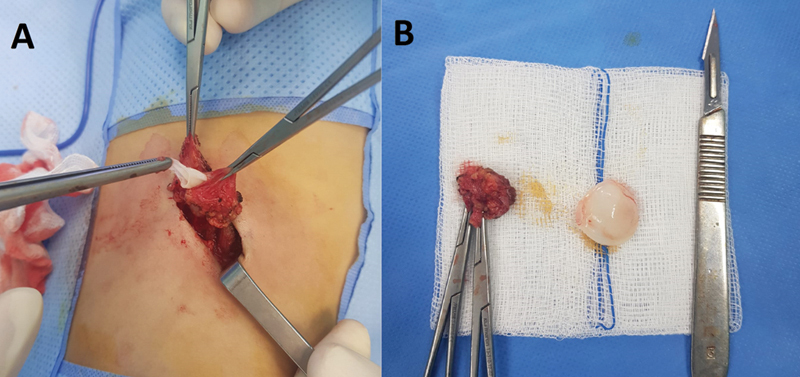
( **A** ) Operative image demonstrating the proligerous membrane within the ruptured cyst. ( **B** ) Postoperative image showing the complete excised cyst.

**Fig. 6:**
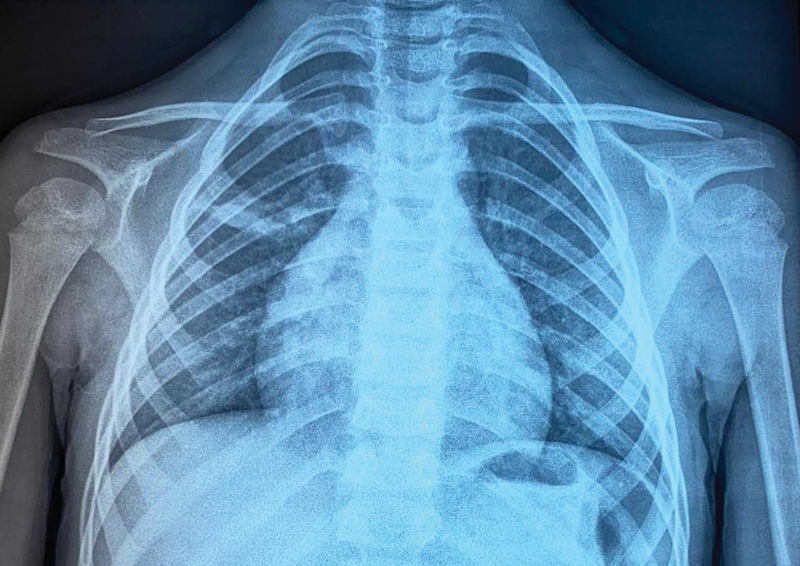
Chest X-ray image after two episodes of hydatidemesis demonstrating a healing of the pulmonary cyst.

**Fig. 7:**
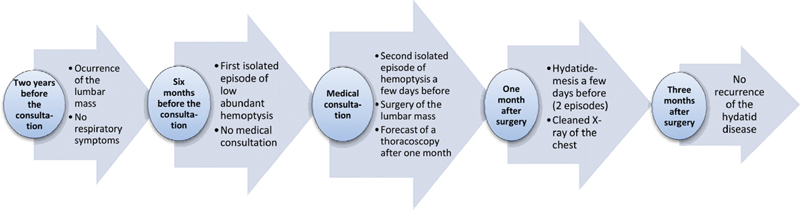
A timeline showing the evolution of the hydatid disease, its medical management and follow-up.

A 6-year-old boy from a rural region was referred to our department of pediatric surgery by the family physician because of two isolated episodes of low abundant hemoptysis with an interval of 6 months. The child was apyretic and in a good general condition. There was no history of surgery, abdominal trauma, vomiting, abdominal or thoracic pain, hematologic disorders, or infectious diseases. Moreover, the patient had no family history of similar cases. Pulmonary vesicular sounds were decreased during auscultation of the right apical thoracic region. On the abdominal examination, a mass on the right lumbar region of 3*1.5 cm (cm) in diameter, firm in consistency, painless, and not fixed to the deeper tissue or to the overlying skin was found that was normal ( Fig. 1 ). It has evolved for 2 years and considered as lipoma at previous medical consultations. On hematological analysis, hemoglobin was 12 g/dL, total leukocyte count was 9120/µL, eosinophil count was within the normal range, and C-reactive protein was 0.55 mg/L. All other biochemical serum parameters were without abnormalities. The hydatid serology was negative. Chest X-ray showed a right middle pulmonary opacity ( Fig. 2 ). Importantly, computed tomography (CT) scan of the chest revealed a fluid density lesion of the right hemithorax, heterogeneous, with irregular contours, not enhanced after injection of the contrast product with a periphery that is enhanced, measuring 39*40*28* mm in size ( Fig. 3A and B ). The abdominal CT scan supplemented by an ultrasound imaging showed a well-defined and anechoic cystic mass of the subcutaneous soft tissues of the right posterolateral abdominal wall that was enhanced in periphery after injection of the contrast product and measuring 32*23 mm in size ( Fig. 4 ). The other abdominal organs were without any other abnormalities, particularly the liver, the spleen, the kidneys, and the pancreas. An anesthetic consultation was requested. The decision was to perform a surgical excision of the abdominal parietal mass under locoregional anesthesia that may help in diagnosis orientation. Surgical exploration showed a cystic mass of 4 cm in size containing a liquid and characterized by a “clear stone liquid” appearance and a proligerous membrane. The operating field was washed with a scolicidal agent using hypertonic saline solution (10% NaCl) and a complete excision of the cyst was performed ( Fig. 5A and B ). The macroscopic appearance of the mass was in favor of a hydatid cyst and the pathological examination of the specimens confirmed the diagnosis of the hydatid disease later, which helped in suggesting the nature of the pulmonary lesion. The child was discharged the same day of his admission in a satisfactory condition after an uneventful postoperative time. A medical treatment for 3 months was started immediately after surgery (albendazole 10 mg/kg/day). A thoracoscopy after 1 month was scheduled to manage the pulmonary localization, but two episodes of hydatid vomiting occurred before performing it leading to spontaneous disappearance of the pulmonary cyst ( Fig. 6 ). No recurrence of the hydatid disease was detected after 3 months of follow-up and no negative incidents in terms of adherence and tolerability were observed. Taken together, the evolution of the disease in our patient, our management, and follow-up are represented in a timeline ( Fig. 7 ).

## Discussion

Hydatid disease remains endemic in Africa as well as in other regions worldwide particularly in South America, Mediterranean countries, Central Asia, and China. 8 Morocco is one of the endemic countries of the hydatid disease with an overall prevalence of 1.9% in rural areas. 9

The liver is the most affected organ since it is the first and the largest filter of parasitic embryos coming from the intestine through the portal system. 10 The lungs and rarely the other organs are also exposed by the systemic circulation after possible escape from this filter. 10 Multiple organs in hydatid cyst are involved in 10.3% of cases and the association liver-lung is described to be the most occurred (7.7%). 11 It was reported that the incidence of hydatid cysts with uncommon localization represents globally 7% and the cases involving soft tissue reach only 1% in pediatric population. 2 This low prevalence can be explained by muscular contractility and the presence of lactic acid making the growing of the cyst difficult. 12 To the best of our knowledge, only five cases of hydatid cysts involving the abdominal wall in children were reported. Those cases interested the right lower abdominal wall, 3 4 the suprapubic region, 5 the right inguinal, 6 and the lumbar regions. 7

The hydatid cyst is generally asymptomatic or minimally symptomatic. 13 For this reason, it can reach large dimensions. 13 Clinical manifestations are nonspecific and depend on the location, the size of the cyst, and the pressure effect of the slow-growing cyst in the affected organ. 14 In soft tissues, it is usually described by the clinicians as painless and noninflammatory swelling, 14 which is consistent with the clinical course of our patient.

Serological testing can confirm the diagnosis, but its accuracy is not high. 15 The localization of the hydatid cyst can clearly influence the intensity of the serological response of the body. 15 Notably, serology of hydatid disease can be positive in most cases but only in 27% of patients with musculoskeletal location. 11 16 In addition, a significant association between a serological negative test and the rupture of the cyst was previously reported. 11 The hydatid serology of our patient was negative that is in line with previous literature findings.

Chest X-ray, ultrasound, CT, and magnetic resonance imaging may be necessary depending on the organ affected and the stage of the disease to confirm the diagnosis. 12 However, an atypical localization of the hydatid cyst with inconclusive imaging findings can be challenging. 12 The hydatid cyst of the soft tissue could be seen radiologically under various aspects including unilocular cyst, cyst with detached membranes, with daughter cysts or with calcified wall. 12 In our case, the mass was seen by ultrasound and CT as unilocular cyst without detached membranes, daughter cysts, or calcified wall making the diagnosis not evident.

Lipoma, which was the diagnosis considered before the presentation of the patient to our department, the sebaceous cyst, and abscess might mimic a hydatid cyst of soft tissues. 17 For this reason, the hydatid disease should always be kept in mind especially in endemic areas to avoid biopsy or partial excision of the mass. Otherwise, the risk of anaphylaxis or recurrence of the disease after spillage should be considered.

The available therapeutic options to clinicians in term of soft tissue hydatid disease include surgery, chemotherapy, and puncture/aspiration/injection/reaspiration (PAIR). 5 Surgical cystectomy is the basis of the treatment. 18 The goals are to remove completely the cyst and to avoid further contamination caused by spillage of cyst content. 18 In our patient, an accidental opening of the cyst during the dissection was occurred and the contamination was avoided by washing the operating field with scolicidal compounds based on hypertonic saline solution. Until to date, the medical treatment based on benzimidazole compounds (albendazole and mebendazole) is still useful to prevent the recurrence and to reduce the size of the hydatid cysts. 19 20 Importantly, a previous report by Srivastava et al showed in a case of a 14-year-old boy with a hydatid cyst of the right lower abdominal wall, of 11.2 × 3.5 cm in size, with multiple daughter cysts that medical treatment using albendazole as monotherapy may also have a place in the therapeutic strategy of the hydatid disease in addition to surgery. In fact, the cyst disappeared after 14 weeks following the albendazole treatment alone. 3 Furthermore, PAIR can be used for inoperable patients or for those who are reluctant to surgery. 20 However, it is necessary to be accompanied by a medical treatment. 20 In our case, the diagnosis of hydatid disease was considered intraoperatively and the medical treatment was started immediately after surgery. During his last follow-up visit, our patient achieved a complete remission and his family was satisfied with our management strategy.

The diagnosis of hydatid cyst with unusual localization remains challenging even in endemic areas of the disease, but it should be kept in mind to avoid any biopsy or partial resection. A total surgical resection of the cyst is the treatment of choice, which should be combined to benzimidazole drugs to prevent possible recurrence. Other localizations particularly in the liver and lungs should be systematically searched.

## References

[1] McManus D P Gray D J Zhang W Yang Y Diagnosis, treatment, and management of echinococcosis BMJ 2012 344 e3866 10.1136/bmj.e3866Published 2012 Jun 11 22689886

[2] Durakbasa C U Tireli G A Sehiralti V Sander S Tosyali A N Mutus M An audit on pediatric hydatid disease of uncommon localization: incidence, diagnosis, surgical approach, and outcome J Pediatr Surg 2006 41 08 1457 1463 16863854 10.1016/j.jpedsurg.2006.04.024

[3] Srivastava P Gangopadhyay A N Upadhyaya V D Sharma S P Jaiman R An unusual presentation of hydatid cyst in anterior abdominal wall Kathmandu Univ Med J (KUMJ) 2008 6 24 511 513(KUMJ) 19483436 10.3126/kumj.v6i4.1746

[4] Erikci V Hoşgör M Aksoy N Primary abdominal wall hydatid cyst: a case report Turk J Pediatr 2014 56 02 183 185 24911854

[5] Tartar T Bakal U Sarac M Akdeniz I Kazez A Primary urachal hydatid cyst in a child: a case report Iran J Parasitol 2019 14 02 352 355 31543926 PMC6737373

[6] Wani S A Baba A A Bhat N A Hamid R Mufti G N Inguinal hydatid cyst in a child: a rare case report Int J Surg Case Rep 2015 10 236 237 25898284 10.1016/j.ijscr.2015.03.050 PMC4430203

[7] Arslan S Turan C Sezer S Tuna I S Primary lumbar hydatid cyst: a case report Turk J Pediatr 2010 52 05 556 558 21434547

[8] Wen H Vuitton L Tuxun T Echinococcosis: advances in the 21st century Clin Microbiol Rev 2019 32 02 e00075 e18Published 2019 Feb 13 30760475 10.1128/CMR.00075-18 PMC6431127

[9] Chebli H Laamrani El Idrissi A Benazzouz M Human cystic echinococcosis in Morocco: ultrasound screening in the Mid Atlas through an Italian-Moroccan partnership PLoS Negl Trop Dis 2017 11 03 e0005384 10.1371/journal.pntd.0005384Published 2017 Mar 1 28248960 PMC5348040

[10] Hamamci E O Besim H Korkmaz A Unusual locations of hydatid disease and surgical approach ANZ J Surg 2004 74 05 356 360 15144257 10.1111/j.1445-1433.2004.02981.x

[11] Tartar T Bakal U Sarac M Kazez A Laboratory results and clinical findings of children with hydatid cyst disease Niger J Clin Pract 2020 23 07 1008 1012 32620733 10.4103/njcp.njcp_531_19

[12] Polat P Kantarci M Alper F Suma S Koruyucu M B Okur A Hydatid disease from head to toe Radiographics 2003 23 02 475 494, quiz 536–537 12640161 10.1148/rg.232025704

[13] Balci A E Eren N Eren S Ulkü R Ruptured hydatid cysts of the lung in children: clinical review and results of surgery Ann Thorac Surg 2002 74 03 889 892 12238856 10.1016/s0003-4975(02)03785-2

[14] Ousadden A Elbouhaddouti H Ibnmajdoub K H Mazaz K Aittaleb K A solitary primary subcutaneous hydatid cyst in the abdominal wall of a 70-year-old woman: a case report J Med Case Reports 2011 5 270 10.1186/1752-1947-5-270Published 2011 Jul 2 PMC3152916 21722386

[15] Zarzosa M P Orduña Domingo A Gutiérrez P Evaluation of six serological tests in diagnosis and postoperative control of pulmonary hydatid disease patients Diagn Microbiol Infect Dis 1999 35 04 255 262 10668582 10.1016/s0732-8893(99)00079-6

[16] Arazi M Erikoglu M Odev K Memik R Ozdemir M Primary echinococcus infestation of the bone and muscles Clin Orthop Relat Res 2005 432 234 241 15738827 10.1097/01.blo.0000149816.86222.2d

[17] Gulmez M Celik A S Alkan S Koban B U Onal R S Uzun M A Primary subcutaneous cyst hydatid of abdominal wall: a case report North Clin Istanb 2015 2 02 152 154Published 2015 Sep 25 28058357 10.14744/nci.2015.58066 PMC5175094

[18] Sayek I Tirnaksiz M B Dogan R Cystic hydatid disease: current trends in diagnosis and management Surg Today 2004 34 12 987 996 15580379 10.1007/s00595-004-2830-5

[19] Dehkordi A B Sanei B Yousefi M Albendazole and treatment of hydatid cyst: review of the literature Infect Disord Drug Targets 2019 19 02 101 104 29956639 10.2174/1871526518666180629134511

[20] Saimot A G Medical treatment of liver hydatidosis World J Surg 2001 25 01 15 20 11213151 10.1007/s002680020003

